# Supplementary material for: Impaired capillary–venous drainage contributes to gliosis and demyelination in mouse white matter during aging
Source: Nat Neurosci. 2025 Aug 12;28(9):1868–82. doi: 10.1038/s41593-025-02023-z (PMC12411274; doi:10.1038/s41593-025-02023-z)
Supplement: Supplementary file 2 — Reporting Summary [file 41593_2025_2023_MOESM2_ESM.pdf]

nature portfolio

Corresponding author(s): Andy Y. Shih, Ph.D.

Last updated by author(s): May 30,2025

Reporting Summary

Nature Portfolio wishes to improve the reproducibility of the work that we publish. This form provides structure for consistency and transparency in reporting. For further information on Nature Portfolio policies, see our Editorial Policies and the Editorial Policy Checklist.

Statistics

For all statistical analyses, confirm that the following items are present in the figure legend, table legend, main text, or Methods section.

n/a	Confirmed	
☐	☒	The exact sample size (n) for each experimental group/condition, given as a discrete number and unit of measurement
☐	☒	A statement on whether measurements were taken from distinct samples or whether the same sample was measured repeatedly
☐	☒	The statistical test(s) used AND whether they are one- or two-sided<br>*Only common tests should be described solely by name; describe more complex techniques in the Methods section.*
☐	☒	A description of all covariates tested
☐	☒	A description of any assumptions or corrections, such as tests of normality and adjustment for multiple comparisons
☐	☒	A full description of the statistical parameters including central tendency (e.g. means) or other basic estimates (e.g. regression coefficient) AND variation (e.g. standard deviation) or associated estimates of uncertainty (e.g. confidence intervals)
☐	☒	For null hypothesis testing, the test statistic (e.g. F, t, r) with confidence intervals, effect sizes, degrees of freedom and P value noted<br>*Give P values as exact values whenever suitable.*
☒	☐	For Bayesian analysis, information on the choice of priors and Markov chain Monte Carlo settings
☐	☒	For hierarchical and complex designs, identification of the appropriate level for tests and full reporting of outcomes
☐	☒	Estimates of effect sizes (e.g. Cohen's d, Pearson's r), indicating how they were calculated

Our web collection on statistics for biologists contains articles on many of the points above.

Software and code

Policy information about availability of computer code

Data collection	In vivo two-photon imaging data was collected with a Bruker Investigator multi-photon microscope running PrairieView software (version 5.5). In vivo three-photon imaging was collected with a modified MIMMS three-photon microscope (Sutter Instruments) running a custom version of ScanImage software (MBF Bioscience).<br><br>Light-sheet data was collected with a SmartSPIM light-sheet microscope (LifeCanvas Technologies).<br><br>Histological data was collected with an Olympus VS120 slide scanner or an Evident Scientific APX100 and viewed with OlyVIA 2.9.1 software. Confocal images were taken with a Zeiss 710 LSM confocal microscope running Zen 2011 software.
Data analysis	All data analysis for in vivo imaging was performed with MATLAB (version R2021a), ImageJ/FIJI (version 1.54f), Imaris x64 (version 10.0), SPSS (version 20) or GraphPad Prism (version 9) software.<br><br>Stitching of the light-sheet fluorescence microscope datasets was done based on a custom code built by the Yongsoo Kim lab; DOI: 10.5281/zenodo.15485079.<br><br>Custom codes for analysis of in silico data are provided through repositories described in Software Policy document. Further details on analysis procedures not described in the Methods are available upon request. In silico data was visualized with Paraview software (version 5.7.0) performed using Python (version 2.7). Further details and explanations of in silico data are available from F.S. upon request.

For manuscripts utilizing custom algorithms or software that are central to the research but not yet described in published literature, software must be made available to editors and reviewers. We strongly encourage code deposition in a community repository (e.g. GitHub). See the Nature Portfolio guidelines for submitting code & software for further information.

Data

Policy information about availability of data

All manuscripts must include a data availability statement. This statement should provide the following information, where applicable:

- Accession codes, unique identifiers, or web links for publicly available datasets
- A description of any restrictions on data availability
- For clinical datasets or third party data, please ensure that the statement adheres to our policy

Raw image files are stored on servers at Seattle Children's Research Institute owing to their large size. Raw image files used in making all figures, along with details of data sampling within these images, will be available upon request from the corresponding author. All source data, such as extracted metrics of vascular structure and blood flow used in figures, are provided as supplementary materials. There are no restrictions on these data.

Research involving human participants, their data, or biological material

Policy information about studies with human participants or human data. See also policy information about sex, gender (identity/presentation), and sexual orientation and race, ethnicity and racism.

Reporting on sex and gender	N/A
Reporting on race, ethnicity, or other socially relevant groupings	N/A
Population characteristics	N/A
Recruitment	N/A
Ethics oversight	N/A

Note that full information on the approval of the study protocol must also be provided in the manuscript.

Field-specific reporting

Please select the one below that is the best fit for your research. If you are not sure, read the appropriate sections before making your selection.

- [x] Life sciences
- [ ] Behavioural & social sciences
- [ ] Ecological, evolutionary & environmental sciences

For a reference copy of the document with all sections, see nature.com/documents/nr-reporting-summary-flat.pdf

Life sciences study design

All studies must disclose on these points even when the disclosure is negative.

Sample size

Sample sizes meet the standard sample size put forth by our prior studies using similar approaches and other groups doing similar experiments. No statistical methods were used to predetermine sample size.

Data exclusions

Out of 12 adult chronic cranial windows, 1 became unusable before the line scan data for layer 6/CC could be obtained. Out of 12 aged windows 1 became unusuable before the line scan data for layer 6/CC could be obtained, while for another only the line scan data for layer 6/CC was obtained. This resulted in 12 complete data sets for layers 2/3 and 4, and 11 complete data sets for layer 6/CC in the adult group, as well as 11 complete data sets for all layers in the aged group.

For analysis of structural and functional parameters of vascular segments, if the signal quality did not allow for reliable analysis of lumen diameter, RBC flux or blood flow velocity these parameters were excluded from the analysis for the corresponding vessel segment, while the length and tortuosity data were included. Correlation analyses were performed only on the population of vessels where all analyzed structural and functional parameters could be obtained.

For 3P capillary ablation experiments performed in awake mice, if a movement or imaging artifact made it impossible to measure a vessels diameter reliably, that vessel was excluded from analysis.

For the UCCAS model experiments. One animal (Mouse 1) died 5 days post-surgery and therefore only baseline and Day 3 post-surgery data are reported. Another mouse (Mouse 5) experienced considerable vascular changes between Days 14 and 21 post-surgery that prevented further data collection. For this mouse, day 21 data was omitted.

Replication

We replicated the results on adult and aged mice imaged under isoflurane anesthesia. The replication cohorts were started with new breeders from Jax, and the experiments were performed approximately a year and a half after collecting data from the initial cohort. Experiments were performed at the Seattle Children's Research Institute by SS, with replication by authors SS and NW.

We also replicated histology results on adult and aged mice. The first batch of experiments was performed by authors SB and MS on a batch

of mice that was previously used for in vivo two-photon imaging experiments under isoflurane anesthesia. The second batch of experiments was performed on a separate cohort of mice, 3 years later by author GG, and included the addition of the mid-aged age group. Both batch of experiments were performed at the Seattle Children's Research Institute.

Our attempts at replicating key study findings using different batches of mice and between different investigative team members were successful.

Randomization

No formal randomization was used for selecting animals for surgery and imaging. However, mice were distributed to the best of our ability with regard to sex and litter of origin in different experimental groups, to reduce influence of these potential covariates. Aging studies present some challenge to balancing sex per age group, as fewer Thy1-YFP females survived to 20-22 months in our studies.

Blinding

Surgeries were performed and in vivo imaging data were collected in an unblinded manner because the experimental groups were readily apparent (i.e. young versus aged mice). However, all analyses were performed blinded to the conditions (e.g. age groups).

Reporting for specific materials, systems and methods

We require information from authors about some types of materials, experimental systems and methods used in many studies. Here, indicate whether each material, system or method listed is relevant to your study. If you are not sure if a list item applies to your research, read the appropriate section before selecting a response.

Materials & experimental systems

n/a	Involved in the study	
☐	☒	Antibodies
☒	☐	Eukaryotic cell lines
☒	☐	Palaeontology and archaeology
☐	☒	Animals and other organisms
☒	☐	Clinical data
☒	☐	Dual use research of concern
☒	☐	Plants

Methods

n/a	Involved in the study	
☒	☐	ChIP-seq
☒	☐	Flow cytometry
☒	☐	MRI-based neuroimaging

Antibodies

Antibodies used

For immunohistochemistry:
Polyclonal rabbit anti-Iba1 (Wako Chemicals 019-19741);
Monoclonal mouse anti-GFAP (Santa Cruz, 2E1);
Monoclonal mouse anti-MBP (Biolegend, SMI 99);
Goat anti-rabbit Alexa Fluor 488 (Invitrogen, A-11008);
Goat anti-mouse Alexa Fluor 488 (Invitrogen, A32723);
Monoclonal mouse anti-GFAP (Sigma-Aldrich, C9205-2ML);
Monoclonal rat anti-MBP (Abcam, ab7349);
Monoclonal rat anti-VCAM1 (Biolegend, 105701);
Polyclonal rabbit anti-pimonidazole (Hypoxyprobe, PAB2627);

Immunolabeling for light-sheet imaging:
Polyclonal rabbit anti-α-SMA (Abcam, ab5694) RRID:AB_2223021, dilution 1:1000;
Polyclonal rabbit anti-transgelin (Sm22) (Abcam, ab14106) RRID:AB_443021, dilution 1:1500;
DyLight-594 labeled Lycopersicon Esculentum (Tomato) Lectin (Vector labs, DL-1177-1), dilution 1:100;
Polyclonal goat anti-PDGFRβ (R&D Systems, AF1042) RRID:AB_2162633, dilution: 1:100;
Polyclonal goat anti-Mouse Aminopeptidase N/CD13 (R&D Systems, AF2335) RRID:AB_2227288, dilution: 1:100;
Alexa Fluor 488- AffiniPure Fab Fragment Donkey Anti-Rabbit IgG (H+L) (Jackson ImmunoResearch laboratories; 711-547-003) RRID:AB_2340620, dilution 1:500;
Alexa Fluor 647-AffiniPure Fab Fragment Donkey Anti-Goat IgG (H+L) (Jackson ImmunoResearch laboratories, 705-607-003) RRID:AB_2340439, dilution 1:500.

Validation

All primary antibodies have been validated by the corresponding manufacturers and cited extensively:
Polyclonal rabbit anti-Iba1 (Wako Chemicals 019-19741) - https://labchem-wako.fujifilm.com/us/product/detail/W01W0101-1974.html
Monoclonal mouse anti-GFAP (Santa Cruz, 2E1) - https://www.scbt.com/p/gfap-antibody-2e1
Monoclonal mouse anti-MBP (Biolegend, SMI 99) - https://www.biolegend.com/en-us/products/purified-anti-myelin-basic-protein-antibody-11469
Monoclonal mouse anti-GFAP (Sigma-Aldrich, C9205-2ML) - https://www.sigmaaldrich.com/US/en/product/sigma/c9205?srsltid=AfmBOorcwGyn5a8dHEfFeC8nQgH6GoRPOOFem6ZN6JXrJ6k1YIZ8e1GY
Monoclonal rat anti-MBP (Abcam, ab7349) - https://www.abcam.com/en-us/products/primary-antibodies/myelin-basic-protein-antibody-12-ab7349?srsltid=AfmBOop9kLWRIdHNGGTuOoA3mDq5MU6qS7-CQXDtPC8Qiw8JG_gbjszT
Monoclonal rat anti-VCAM1 (Biolegend, 105701) - https://www.biolegend.com/en-us/products/purified-anti-mouse-cd106-antibody-139
Polyclonal rabbit antipimonidazole (Hypoxyprobe, PAB2627) - chrome-extension://efaidnbmnnnibpcajpcglclefindmkaj/https://site.hypoxyprobe.com/knowledge-center-articles/HP-PAb2627-Antibody-Insert-2020.pdf
Polyclonal rabbit anti- α-SMA (Abcam, cab5694) - https://www.abcam.com/products/primary-antibodies/alpha-smooth-muscle-

actin-antibody-ab5694.html
Polyclonal rabbit anti-transgelin (Sm22) (Abcam, ab14106) - https://www.abcam.com/products/primary-antibodies/taglntransgelin-antibody-ab14106.html
DyLight-594 labeled Lycopersicon Esculentum (Tomato) Lectin (Vector labs, DL-1177-1) - https://vectorlabs.com/products/dylight-594-lycopersicon-esculentum-tomato
Polyclonal goat anti-PDGFRβ (R&D Systems, AF1042) - https://www.rndsystems.com/products/mouse-pdgf-rbeta-antibody_af1042
Polyclonal goat anti-Mouse Aminopeptidase N/CD13 (R&D Systems, AF2335) - https://www.rndsystems.com/products/mouse-aminopeptidase-n-cd13-antibody_af2335

Animals and other research organisms

Policy information about studies involving animals; ARRIVE guidelines recommended for reporting animal research, and Sex and Gender in Research

Laboratory animals	The in vivo two-photon imaging, histology and qPCR experiments were performed on Thy1-YFP mice bred on the C57Bl/6 background (B6.Cg-Tg(Thy1-YFP)HJrs/J; Jax ID 003782). Age group ranges were determined by the Jax "Life Span as a Biomarker" criteria, which defines 3-6 months as "mature adult" stage, and 18-24 months as "old" stage in mice. In vivo three-photon imaging and capillary ablation as well as UCCAS model experiments were performed on adult (5-7 months old) and C57Bl/6 mice. Light-sheet imaging experiments were performed on adult (2 months old) and aged (24 months old) C57Bl/6 mice.
Wild animals	No wild animals were used in this study.
Reporting on sex	Male and female F1 offspring were used across all groups, from ages 5-7 months, and 22-24 months. For in vivo two-photon imaging experiments the adult group had an equal mixture of male and female mice (6 per gender), while the aged group has a slightly higher number of male mice (9 male and 3 female mice). The effect of gender on analyzed parameters was included as a covariate in the statistical analysis for a limited set of experimental variables (pre-convergence capillary RBC flux and diameter).
Field-collected samples	No field samples were collected in this study.
Ethics oversight	All procedures in this study were approved by the Institutional Animal Care and Use Committee at the Seattle Children's Research Institute and the Allen Institute. Both institutions have accreditation from the Association for Assessment and Accreditation of Laboratory Animal Care (AAALAC), and all experiments were performed within guidelines.

Note that full information on the approval of the study protocol must also be provided in the manuscript.

Plants

Seed stocks	N/A
Novel plant genotypes	N/A
Authentication	N/A
